# Computational guided identification of novel anti-mycobacterial agent proved by in-vitro and in-vivo validation

**DOI:** 10.1186/s12866-025-04361-1

**Published:** 2025-09-18

**Authors:** Mohab M. Shalaby, Reham Samir, Kareem A. Ibrahim, Tharwat R. Elkhamissy, Mohammed A. Rammadan

**Affiliations:** 1 Microbiology & Immunology Department, Faculty of Pharmacy, Egyptian Russian University, Cairo, Egypt; 2 Microbiology & Immunology Department, Faculty of Pharmacy, Cairo University, Cairo, Egypt

**Keywords:** *Mycobacterium*, Tuberculosis, Maleic acid, Isoniazid, Computational analysis, DEG

## Abstract

**Background:**

An upsurge of antibiotic resistant bacteria such as *Mycobacterium tuberculosis* is recorded on daily bases as a result of many factors including: the daily antibiotics exploitation, failure to follow lengthy complex drug regimen, and ongoing bacterial mutation. TB treatment protocol is usually a lengthy and expensive one that is composed of 4 or even 5 drugs that have multiple substantial side effects. Traditional drug discovery methodologies are usually lengthy multifaceted process complicated with unpredictable outcomes in terms of efficacy and safety, hence there is an urge to find innovative drug discovery method that can produce multiple novel potential antimycobacterial agents that are safe and effective both in-vitro and in-vivo.

**Results:**

The obtained results illustrated that maleic acid represented a potential drug with minimum inhibitory concentration of 312 µg/ml and an identical minimum bactericidal concentration against *Mycobacterium tuberculosis*. Its IC50 was measured to be 374.44 mg/ml with SI of 1200. Preliminary testing showed that maleic acid can be considered as a possible histidinol-phosphate aminotransferase inhibitor with a high binding affinity (-5.0475 kcal/mol) and promising molecular dynamics. Maleic acid combination with rifampicin had ƩFIC of 0.375 which indicated synergistic activity between them. It efficiently produced 3 ± 0.3009 log_10_ CFU reduction of infected mice lungs compared to control group and illustrated superior preservation of lung tissue and structure on histological screening level.

**Conclusion:**

After careful filtration processes, computational guided scavenge of online protein databases for potential druggable targets represents a promising pathway for identification of novel antimycobacterial agents. One of the promising identified agents was maleic acid which can act as an alternative/additional drug for combating tuberculosis infection.

**Supplementary Information:**

The online version contains supplementary material available at 10.1186/s12866-025-04361-1.

## Introduction

Respiratory infections pose a significant global health challenge due to their potentially life-threatening nature. They are highly diverse in both severity (ranging from acute to chronic) and etiology, which may be viral, bacterial, fungal, or even parasitic. These infections are widespread across all age groups, from pediatric to geriatric populations, largely due to their ease of transmission through respiratory droplets [1]. While the spotlight has been on COVID-19 disease caused by SARS-CoV-2 virus since its emergence in 2019, and continues to some extent, tuberculosis (TB), a bacterial respiratory infection, remains the second deadliest respiratory disease globally [2]. TB is a slow-progressing, chronic infection with a complex prognosis and treatment course. It is transmitted mainly through respiratory aerosols, such as those expelled during coughing, and spreads easily in both community and healthcare settings. Immunocompromised and malnourished individuals are at greater risk of contracting the disease. At any time point, around one quarter of human population is infected with TB (active, dormant or reservoir) with novel millions of infections each year [3], WHO reported that ≈ 7.5 million additional patients were infected in 2022, this number surpasses the highest infection peak in 2019 (7.1 million novel infections) and is considered the highest since the beginning of WHO’s TB monitoring program. Whereas pulmonary TB maintains the highest fame, other forms of TB (Miliary TB) exist with a real life threatening implications, for instance in 2022 TB mortality was ≈ 1.3 million death that is considered around double that of HIV/AIDS [4]. If left untreated TB mortality percentage can reach 50% of infected cases. Whereas treatment protocols approved by WHO can theoretically decrease this percentage by ≈ 35%, The actual decrease is much reduced since those protocols are usually lengthy (a 4 to 6 month protocol) with the administration of multiple agents along with other influencing factors such as poverty accompanied by its consequent malnutrition in developing countries, and immunocompromised populations such as those suffering from HIV infection, diabetes, and smoking [5–7].

The incoherent compliance to treatment protocols along with unceasing mutations have led to upsurge of resistant *Mycobacterium* species. According to WHO about 410,000 individuals tested positive for multidrug-resistant, rifampicin resistant, or extensively resistant TB (MDR/RR-TB/XDR-TB respectively) in 2022 which is approximately double the recorded number in 2019 [2]. Traditional anti-mycobacterial agents (AMA) discovery techniques are usually hindered and decelerated by: slow growth nature of *Mycobacterium tuberculosis (M. tuberculosis)*, the lengthy complex processes to either synthesize novel AMA or modifying existing drugs (either to enhance their activity or overcome resistance), and the limitations of synthesized/modified agents in terms of their biosafety profile and/or in-vivo efficacy [8–10], this calls for rigorous work to develop and validate novel drug discovery techniques that put the focus on *M. tuberculosis* proteome to produce novel treatment options combating TB. In our work a novel time saving drug discovery strategy was trialed that would produce several potential drugs and possessed ease of access. This strategy operated through 3 consecutive pathways: the 1 st pathway implemented the principles of computational based subtractive proteomic analysis of the *M. tuberculosis* proteins [11, 12] in order to recover potential therapeutic targets, the 2nd pathway utilized *Mycobacterium smegmatis* (*M. smegmatis*) a rapid growing *mycobacterium* as a surrogate to *M. tuberculosis to save time* [13] and finally, the 3rd pathway was to characterize obtained drugs by subjecting them to multiple in-vitro and in-vivo validation tests against *M. tuberculosis*.

## Methods

### Computational guided scavenge for potential drug targets

Numerous online databases and tools were used to reach promising outcomes in terms of an AMA. The focus was to retrieve essential *M. tuberculosis* proteome components from 3 different databases and then subjecting them to a sequential number thinning filtration process to come out with an optimum result.

#### Online databases scavenge for M. tuberculosis proteome

A total of 3 online microorganism’s proteome databases were selected from multiple options based on diversity, conventionality, and time saving. The first selected database was database of essential genes (DEG) [14, 15] which explicitly provides essential proteins; Virulence factor database (VFDB) [16, 17] which provides the joint Virulence factors of many species within the same genus; and Online Gene Essentiality database (OGEE) [18, 19] which provides the entire organism proteome categorized into essential, non-essential, or conditional. All the available protein sequences were retrieved from the 3 databases and then subjected to the next phase.

#### Essentiality determination of recovered proteins

The whole protein sequences recovered from DEG database were characteristically essential, whereas in case of OGEE database only proteins deemed essential were retrieved and cross checked against *M. tuberculosis* sequences available in DEG database using BLASTP tool, as for the VFDB database all the recovered sequences were tested for essentiality by blasting against DEG database likewise OGEE sequences [20].

#### Determination of subcellular localization

While DEG database provides the subcellular localization for each sequence, both VFDB and OGEE don’t provide such information and hence it was determined by using BLASTP tool available at UNIPROT website [21] implementing the following parameters: Matrix used was BLOSUM62 and E-Threshold was 0.0001 [22]. If UNIPROT database didn’t include the data another online tool was implemented to detect localization, this tool was CELLO (subcellular localization predictor) [23], blast criteria used were: selecting protein database and Gram positive bacteria domain [24, 25]. Any extracellular or secreted target was omitted.

#### Homo sapiens proteome similarity inspection

In order to lessen any possible interaction with human proteome all recovered protein sequences were blasted against the human proteome available at National Center for Biotechnology Information (NCBI) [26] by implementing the following rules: Matrix used was BLOSUM62, E-Threshold was 0.0001, Organism was *Homo sapiens* (taxid: 9606), and used database was non-redundant protein sequences (nr) [27]. Any sequence that showed any degree of similarity was disqualified from the list.

#### Inspecting protein preservation in various M. tuberculosis strains and other Mycobacterium species

The universality of recovered proteins among *M. tuberculosis* strains and other *Mycobacterium* species (specifically *M. smegmatis*) was crucial to ensure potential drug effectiveness, and hence the protein sequences were blasted against multiple strains and *M. smegmatis* to ensure its persistent presence in a wide array of strains. NCBI BLASTP was once more utilized implementing the same parameters under Sect. 2.1.4 with the exception of altering the organism from *Homo sapiens* to the following organisms: *M. tuberculosis* complex (taxid:77643), *M. tuberculosis* (Zopf 1883) Lehmann and Neumann 1896 (taxid:1773), *M. tuberculosis* var. hominis (taxid:1773), *M. tuberculosis typus gallinaceus* (taxid:1764), *M. tuberculosis var. bovis* (taxid:1765), *M. tuberculosis subsp. canetti* (taxid:78331), and *M. smegmatis* MC2 155 (taxid:246196) [28].

#### Establishing targets affinity to bind to potential drugs

The final list of targets were eventually tested for the possibility of binding to any of the drugs available at the DRUGBANK [29] implementing the default parameters at the database with a mild modification of setting E-value at 0.0001. The search was conducted against all databases of drug types [20, 30].

### Bacterial strains

*M. tuberculosis* H37Rv ATCC 27,294, *M. smegmatis* mc2 155 ATCC, and *Bacillus cereus* NCTC 2599 standard strains were obtained from Microbiological Resource center Mircen^®^, while *Staphylococcus aureus* ATCC 25,737 and *Escherichia coli* ATCC 25,922 were available at our laboratory. *Klebsiella pneumoniae* KB700603 was kindly donated to us. Clinical samples were obtained from Animal Health Research Institute at Dokki - Egypt and were tagged as MtS1, MtS2, and MtS3.

### Minimum inhibitory concentration (MIC) Pinpointing for potential AMA

Pinpointing minimum inhibitory concentration (MIC) for *M. tuberculosis* can be a tedious time-consuming process. In terms of time saving, drug testing and MIC identification were primarily carried out against *M. smegmatis* then when a drug showed potential, was thereafter tested against *M. tuberculosis*. This was possible since *M. smegmatis* was presented as a surrogate to *M. tuberculosis* in terms of testing potential drugs and genetic wise [13, 31, 32].

#### Inoculum preparation

A stock suspension of any *Mycobacterium* strain was simply prepared by suspending obtained cell pellet from a fresh culture into sterile saline. To achieve homogenous suspension 7–10 sterilized glass beads (3.5–4.5 mm in diameter) were added to each falcon tube to ensure breaking down of bacterial cells lumps and uniform suspension of cells while vortexing at adequate speed [33].

Obtained bacterial suspension was then diluted till reaching a final concentration of 0.5 McFarland approximately ≈ 1 × 10^6^ CFU/ml, which served as a stock suspension. For the purpose of preparing the inoculation suspension, a 1 ml Aliquot from the 0.5 McFarland was diluted10-times so as to reach an inoculum density of ≈ 1 × 10^5^ CFU/ml [34].

#### Preparation of potential AMA

Each AMA was dissolved in its respective solvent so as to reach a stock solution of 10,000 µg/ml.

#### MIC Pinpointing for M. smegmatis implementing broth microdilution technique

Assessment of MIC for *M. smegmatis* was carried out in accordance with Clinical & Laboratory Standards Institute (CLSI) and European Committee on Antimicrobial Susceptibility Testing (EUCAST) guidelines [33, 35]. Initially Aliquots of 100 µl of double strength cation adjusted Muller Hinton broth (NEOGEN^®^, USA) were dispensed into each well, afterwards a 100 µl of the tested agent stock (10,000 µg/ml) was added to the first well in the row and serially twofold diluted by transferring 100 µl into the next well till reaching the 10th well. Eventually a 10 µl of inoculation suspension was added to each well so as to reach a final concentration of 1 × 10^4^ CFU/ml and plates were incubated at 37℃. A sterile water containing beaker was placed adjacent to plates, and both were partially sealed in a rigid plastic bag so as to prevent plates drying with long incubation period [35]. Each experiment also contained a positive and negative control and was conducted in triplicates.

#### MIC pinpointing for M. tuberculosis implementing agar dilution technique

Multiple dilutions of antimicrobial agents were prepared in Middlebrook 7H11 + OADC (BD DIFCO^®^, USA) so as to obtain final concentrations ranging from 156 up to 5000 µg/ml. Aliquot of 5 ml from each concentration was dispensed into a single compartment of 4 compartment petri dish, hence each plate contained 3 different dilutions along with a control quadrant. Inoculum was added in 100 µl Aliquot to each compartment in the form of 3 separate drops, inoculum was prepared at a concentration of 5 × 10⁵ CFU/ml and experiment was conducted in triplicates [33, 36].

### Pinpointing of minimum bactericidal concentration (MBC) for tested drugs

After MIC pinpointing completion, a 10 µl Aliquot from each well that showed null growth was transferred into a new well in a new plate containing 90 µl normal saline (1/10 dilution to remove any residual antibacterial activity) afterward it was plotted on 7H11 Middlebrook + OADC agar plate. The least dilution that showed no visible colonies at agar plates was credited MBC [37].

### Illustrating potential AMA agents’ effect on human lung cells

Assessment of selected AMA biocompatibility with human fetal lung fibroblast cells (WI-38) were trialed implementing a process similar to [38] with mild adjustment. Briefly 5 sequential concentrations were prepared from AMA: AMA MIC, two sequential 10-fold dilution of MIC, and two sequential 10-fold increments of MIC (3.12-31.2-312-3120-31200 µg/ml). To flat bottomed 96-wells plate a 120 µl of cell suspension was pipetted in every well followed by 30 µl of each drug concentration (except control wells) to reach a final volume of 150 µl ensued by incubation at 37 °C for 2 h, after incubation conclusion wells were double washed with phosphate buffered saline (PBS) (Thermo Fisher Scientific^®^, USA). Thereafter MTT (3-(4,5-dimethythiazol-2-yl)−2,5-diphenyl tetrazolium bromide) (Merck^®^, USA) was added in 15 µl aliquots to each well (10% of total well content), mixed thoroughly for 5 min duration followed by additional incubation for 4 h at 37 °C. Subsequently 150 µl aliquots of acidic isopropanol were pipetted (to induce cell lysis and crystal solubilization) then incubated for 1 h at identical condition as mentioned above. ELISA plate reader was used to measure the developed color produced as a result of intercellular reduction of MTT (yellow) to formazan product (purple) at OD of 570 nm (background wavelength was 630 nm) [39, 40].

### Inspecting selected AMA range of activity

AMA with illustrated potentials were subjected to spectrum of activity determination process via testing them against representatives of Gram positive (Gram + ve) and Gram negative (Gram-ve) bacteria. The tested bacteria included the following: *Staphylococcus aureus* ATCC 25,737 a Gram + ve ESKAPE Pathogen, *Bacillus cereus* NCTC 2599 a Gram + ve pathogenic bacilli (similar to *M. tuberculosis*), *Escherichia coli* ATCC 25,922 a Gram-ve ESKAPE pathogen, *Klebsiella pneumoniae* KB700603: A Gram-ve respiratory ESKAPE pathogen. Tested organisms were subcultured, harvested, and diluted so as to obtain a working suspension of ≈ 1 × 104 CFU/ml then MIC determination was accomplished as previously mentioned (2.3.3.) [41, 42].

### Induction of potential resistance against selected AMA

A methodology similar to [43] with some modification was implemented, briefly after completion of MIC & MBC experiments an Aliquot from the 1st concentration less than MIC was recovered and centrifuged at 4℃ and 9,503 x g for the duration of 10 min., supernatant was subsequently discarded while the pellet was washed twice with 1 ml sterile saline (to remove residual AMA & media) and used as an inoculum for a novel ensuing experiment. *Mycobacterium* species were challenged in this manner up to 20 consecutive times.

### Molecular Docking of maleic acid against histidinol-phosphate aminotransferase

Molecular docking simulation was performed for maleic acid against the Rv1600 encoded aminotransferase: histidinol-phosphate aminotransferase (HisC) using Molecular Operating Environment (MOE), 2019.0102 software [44, 45]. Maleic acid was drawn and prepared via energy minimization, hydrogen addition and calculation of the partial charges. Finally, it was saved in the form of mdb extension [46, 47]. The target isozyme was retrieved from Protein Data Bank (www.rcsb.org) with PDB ID: 4R8D. It was prepared and checked through automatic quick prepare order of MOE. The docking simulation was implemented via Amber10 Forcefield. Evaluation of ligand-protein complex interactions was afforded through visualization of poses and scoring function [48, 49]. Validation of docking study was examined by the Root Mean Square Deviation (RMSD) value for co-crystalized ligand protein isozyme seemed to be equal to 1.4054.

### Molecular dynamic simulation

The MD simulations were carried out by iMod server (iMODS) (http://imods.chaconlab.org) which gives a convenient interface for this enhanced normal mode analysis (NMA) methodology in inner coordinates [50] at 300 K constant temperature and 1 atm constant pressure.

### Preliminary confirmation of HisC Inhibition by maleic acid

Kyoto Encyclopedia of Genes and Genomes (KEGG) database was scanned to retrieve the enzymatic reaction, substrates, and products, of HisC enzyme (EC:2.6.1.9) a potential drug target. Glutamic acid was identified as one of the reaction main products. Inhibition of HisC enzyme would mean prevention of glutamate formation and hindrance of histidine pathway. In theory, external addition of glutamic acid to bacterial media would salvage HisC inhibition by providing the reaction product in ready form for the next reaction in histidine pathway, and hence restore *M. tuberculosis* survival. Since maleic acid came out as a potential drug acting on HisC enzyme proposed by DRUGBANK, The actual inhibition of HisC by maleic acid was evaluated by providing the MIC test mixture with increasing concentrations of glutamic acid (Merck^®^, Germany), if maleic acid MIC increased with the addition of glutamic acid, this would indicate overcoming of HisC inhibition and then this can be a preliminary confirmation of possible inhibition of HisC by maleic acid. Glutamic acid was added to each well in 100 µl Aliquots at a concentration ranging from 134 µg/ml to 4300 µg/ml [51, 52].

### Potential synergism assessment between AMA and standard anti-mycobacterium drugs

Inoculum was prepared as previously mentioned (2.3.1) while AMA was prepared at 2 different concentrations; 10,000 µg/ml and 20,000 µg/ml which were dubbed M1 and M2, respectively. Anti-tuberculous drugs isoniazid and rifampicin were selected from the standard treatment protocol and were prepared at a concentration 10 times their MICs and were dubbed I & R, respectively.

A procedure similar to [53] was implemented. In brief, 100 µl of double strength cation adjusted Muller Hinton broth was added to each well in a 96 well microtiter plate with curved bottom, 100 µl of M1 were added to wells A2 – A12 then serially diluted till row G (excess amount was discarded from row G), 100 µl of M2 was added to well A1 then serially diluted till well G1. At the following step a 100 µl of I Or R were added to wells A1 – H1 then serially diluted till column 11 (excess amount was discarded from column 11), and finally a 10 µl of the prepared inoculum was added to each well except well H12 (which served as a negative control), plates were sealed and incubated as mentioned before. Synergistic activity was determined following 2 approaches: the 1 st by manually calculating ∑FIC value, synergistic activity was recorded when ∑FIC ≤ 0.5, the 2nd approach utilized the online tool SynergyFinder^®^ [54, 55], the following criteria were selected: readout was based on inhibition, outliers were detected, curve fitting was set to LL4, synergism calculation method was set to HSA, synergistic activity was recorded when synergy score was > 10. Results were recorded as %inhibition of cells that was computed utilizing OD reading of each well at 600 nm wavelength and implementing the following Eq. (56).

$${\%}\text{I}\text{n}\text{h}\text{i}\text{b}\text{t}\text{i}\text{o}\text{n}\:=\frac{\text{O}\text{D}\text{c}-\text{O}\text{D}\text{t}}{\text{O}\text{D}\text{c}}\text{X}\:100$$

OD_c_ and OD_t_ represent positive control and test optical densities, respectively.

### In-vivo determination of anti-mycobacterial activity of selected AMA

#### Mice selection and immunosuppression Preparation for infection

Male BALB/c mice were selected for their ease of handling and suitability for rapid evaluation of AMA [56]. Mice were obtained at a weight of 20 ± 2 gm. They were housed at cages (12/cage) and supplied by food and water ad libitum, they were left for 7 days for acclimatization. Following the 7 days and on Day 1 of experiment, mice were administered dexamethasone (Merck^®^, Germany) (prepared in 1X PBS) at a dose of 5 µg/gm via subcutaneous injection every day for 14 days SID except for the day of infection (Day 8) [57–59].

#### M. tuberculosis inoculum Preparation and mice infection

Inoculum preparation for in-vivo infection was prepared according to [60], with the intention of delivering ≈ 1 × 10^2^ CFU/lung; a 0.5 McFarland suspension was prepared as previously mentioned (≈ 4 times the required CFU to be delivered into lungs). All preparations and dilutions were carried out using 1X PBS.

On Day 8, no dexamethasone treatment was administered and intranasal infection was carried out by: partially sedating each mice using sub effective dose (0.5%) of Isoflurane (Merck^®^, Germany), mice were held in vertical position and 40 µl suspension was delivered at 2 equivalent doses drop wise into both nostrils, mice were allowed to breath in 1 st dose drops and left for 2 min before delivering a second one [61]. After infection, mice were left for 24 h without any further agitation, then on Day 9 dexamethasone treatment was recommenced for another 7 days. On Day 9, three mice were euthanized and their lungs were harvested to ensure accurate delivery of ≈ 50–100 CFU/lung.

#### Drugs preparation, mice segregation and treatment commencement

Drugs were weighed and dissolved in 1X PBS then sterilized using 0.22 μm filters tp achieve a concentration equivalent to 10X their MIC against *M. tuberculosis* (3125 and 1 µg/ml for maleic acid and isoniazid, respectively) as a conservative and effective starting concentration in order to achieve at least a 1- log10 CFU reduction as suggested by [62, 63], 40 µl from each drug was administered intranasally (20 µl per nostril) into corresponding mice group to achieve direct local effect. Since mice weighed ≈ 20 gm then the achieved doses were ≈ 6.24 mg/kg of maleic acid and 0.002 mg/kg of isoniazid.

On Day 15, 3 mice were euthanized and harvested lungs were subjected to viable cell enumeration to determine the CFU/lung before treatment (served as pretreatment group). The remaining Mice were administered the last dose of dexamethasone and then were segregated into 4 different groups with 13 mice/group labeled I for isoniazid (Merck^®^, Germany), M for maleic acid (Merck^®^, Germany), P for PBS, and C for control. Group I, M, and P were administered a 40 µl dose of their represented agent, group C wasn’t administered anything and served as a control group. Treatment was maintained SID for 7 days.

On Day 22, 8 mice from each group were euthanized with an overdose of isoflurane then the chest cages were opened and lungs were harvested using sterile scissors and forceps. Harvested lungs were maintained in 1 ml 1X PBS for further examination. After day 22, 5 mice from each group were kept till Day 56 for histopathological analysis.

#### Lungs processing and CFU determination

Lungs were weighed and their weights were recorded. Harvested lungs were homogenized into 1 ml of 1X PBS using Omni Macro Homogenizer (Omni international^®^, USA) till achieving homogeneity. A 10 µl Aliquot from each homogenized lung was inoculated onto Middlebrook 7H10 + OADC plate, then a 20 µl Aliquot form the homogenate was serially diluted by 10 folds till 10^−8^ dilution. Finally, 10 µl aliquot from each dilution was inoculated onto the previously mentioned plate. Incubation was conducted at standard conditions and development of any growth was recorded.

#### Histopathological analysis of lung tissue from various treatment groups

On Day 56, remaining mice were euthanized, their lung were harvested, dissevered lungs tissue from the various groups were flushed then fixed in neutral buffered formaldehyde (10%, v/v) for ≈ 72 h. Samples were subjected subsequently to multiple steps started with trimming followed by processing in serial alcohol grades, then cleared in xylene, and eventually embedded & infiltrated with Paraplast tissue embedding media. Rotatory microtome was utilized to cut 4 μm thick tissue sections with the intention of demonstrating the pulmonary parenchyma then fixing it to glass slides. A stain mixture of hematoxylin and eosin was applied to tissue sections as a mean of examination by staining (for visualization of general morphological characters) submitted to light microscopy-based examination by experienced histologist via the use of full HD microscopic imaging system (Leica Microsystems GmbH, Germany). All standard procedures for samples fixation and staining were according to [64].

### Statistical analysis

All statistical analysis tests were carried out utilizing GraphPad Prism 9.1.0.221, implemented tests included: unpaired t-test and one way ANOVA to detect significant difference between various in-vivo groups, nonlinear regression (curve fit) for IC50 calculation.

All experiments were carried out in triplicates.

## Results

### Protein databases scavenge and filtration

**Fig. 1:**
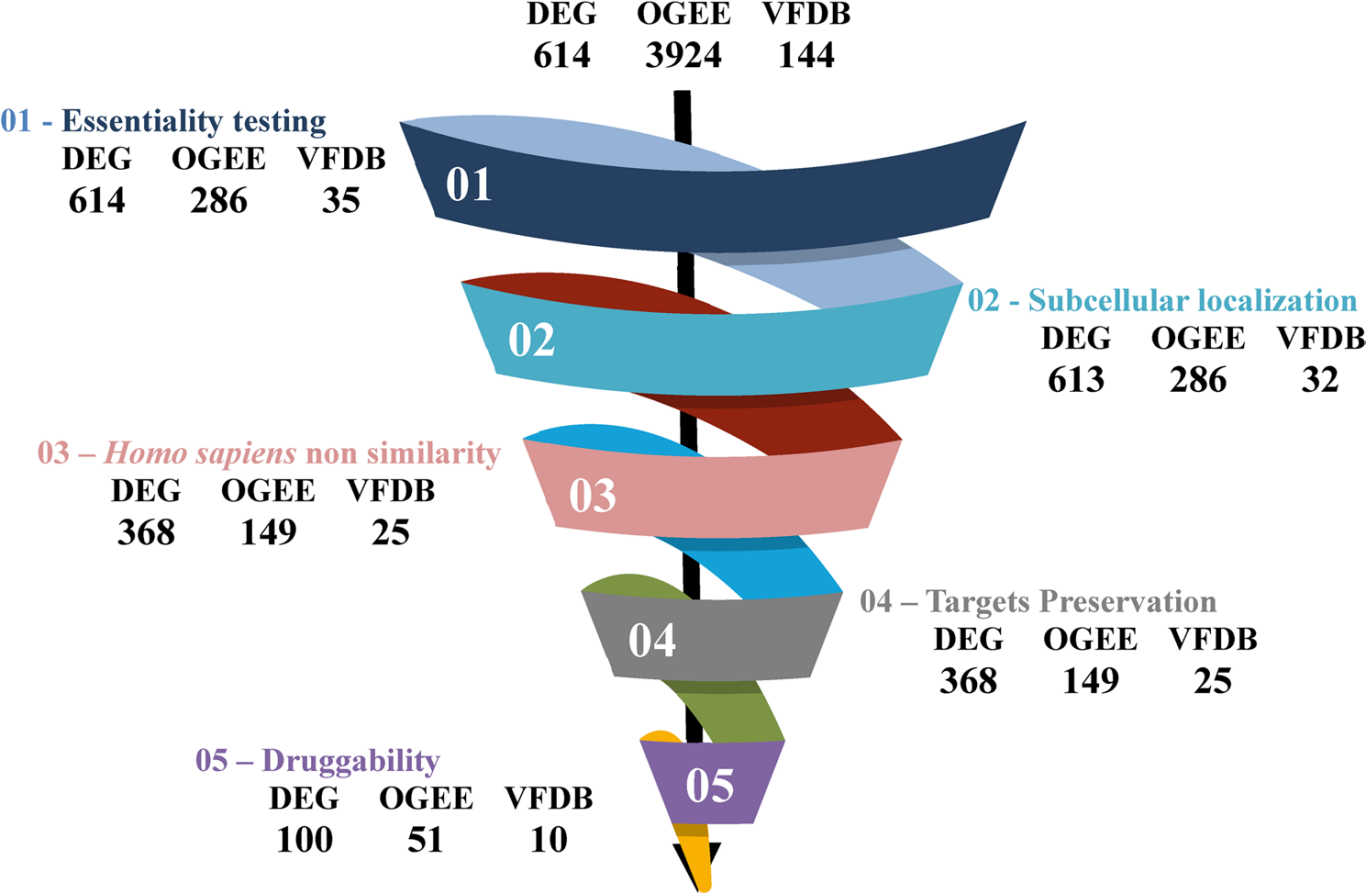
Filtration process diagram with results at each step.The diagram shows the successive filtration processes with the resulting targets after each step for each database. The obtained final targets were 100, 51, and 10 for DEG, OGEE, and VFDB, respectively

The entire protein targets from both DEG, OGEE, and VFDB data bases were pooled for analysis which disclosed a total of 614, 3924 and 144 proteins, respectively. In case of OGEE a classification of proteins according to essentiality readily exist and it’s classified into essential, non-essential, and conditional, hence only proteins deemed essential were retrieved which came down to a total of 286 proteins. The scavenge process for a novel druggable protein targets followed successive filtration steps as shown in Fig. 1, the steps were similar for each database with exception of essentiality testing for DEG (since it’s used as the reference database).

**Fig. 2:**
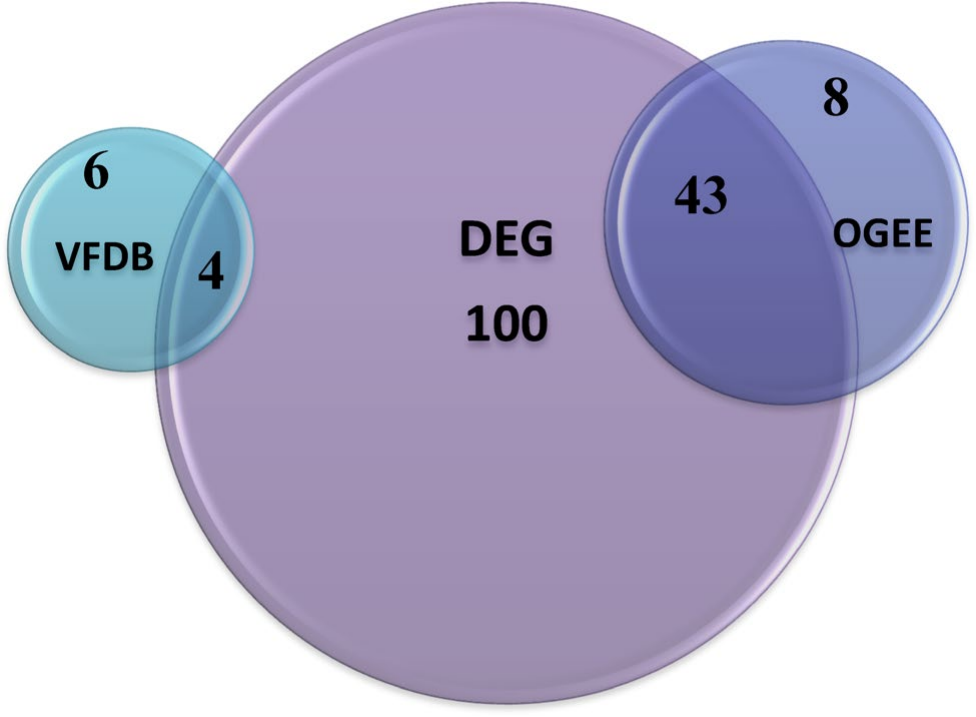
Mycobacterial protein targets comparison and similarity across the 3 databases.A diagram showing that the largest number of protein targets were obtained from DEG database which had 43 & 4 targets in common with OEGG & VFDB, respectively, and 54 unique targets. OGEE & VFDB has 8 & 6 unique targets, respectively

After the successive filtration, a total of 100, 51, 10 targets met the criteria from each database, respectively. For OGEE database, 43 out of 51 targets were already present in DEG database for H37Rv I strain; as per VFDB, 4 out of 10 targets were already present in DEG database for H37Rv I strain as shown in Fig. 2, the remaining targets were present in the database for H37Rv II strain, an additional Excel file shows DEG targets in more detail [see Additional file 1].

### Retrieved targets characteristics

Each database provided multiple targets that after passing through the sequential filtration process yielded fewer essential non-*homo sapiens* similar druggable targets that are mainly cytoplasmic in nature.

As per DEG, a final 100 targets met the criteria above. Following OGEE and VFDB; characteristics of only unique targets (not present in DEG) were detected and reported which exposed only 8 and 6 inimitable targets, respectively that met the criteria. Targets showed the following characteristics as shown in Table 1.

**Table 1:** Basic characteristics of mycobacterial retrieved targets from different databases

	Subcellular localization				DNA strand localization		Targets length (aa)	
Databases	Cytoplasmic	Plasma membrane	periplasmic	unknown	Leading	Lagging	Max	Min
*DEG*	78	8	3	11	77	23	1527	73
*OGEE*	7	1	-	-	6	2	402	22
*VFDB*	4	2	-	-	4	2	1004	139

The table shows basic information about retrieved targets such target subcellular localization, DNA strand localization of these targets and the size of the smallest vs. the largest retrieved protein. Only inimitable targets from both OGEE & VFDB were included in the analysis. *aa* amino acid

### Categorization of obtained drugs from all targets

**Fig. 3:**
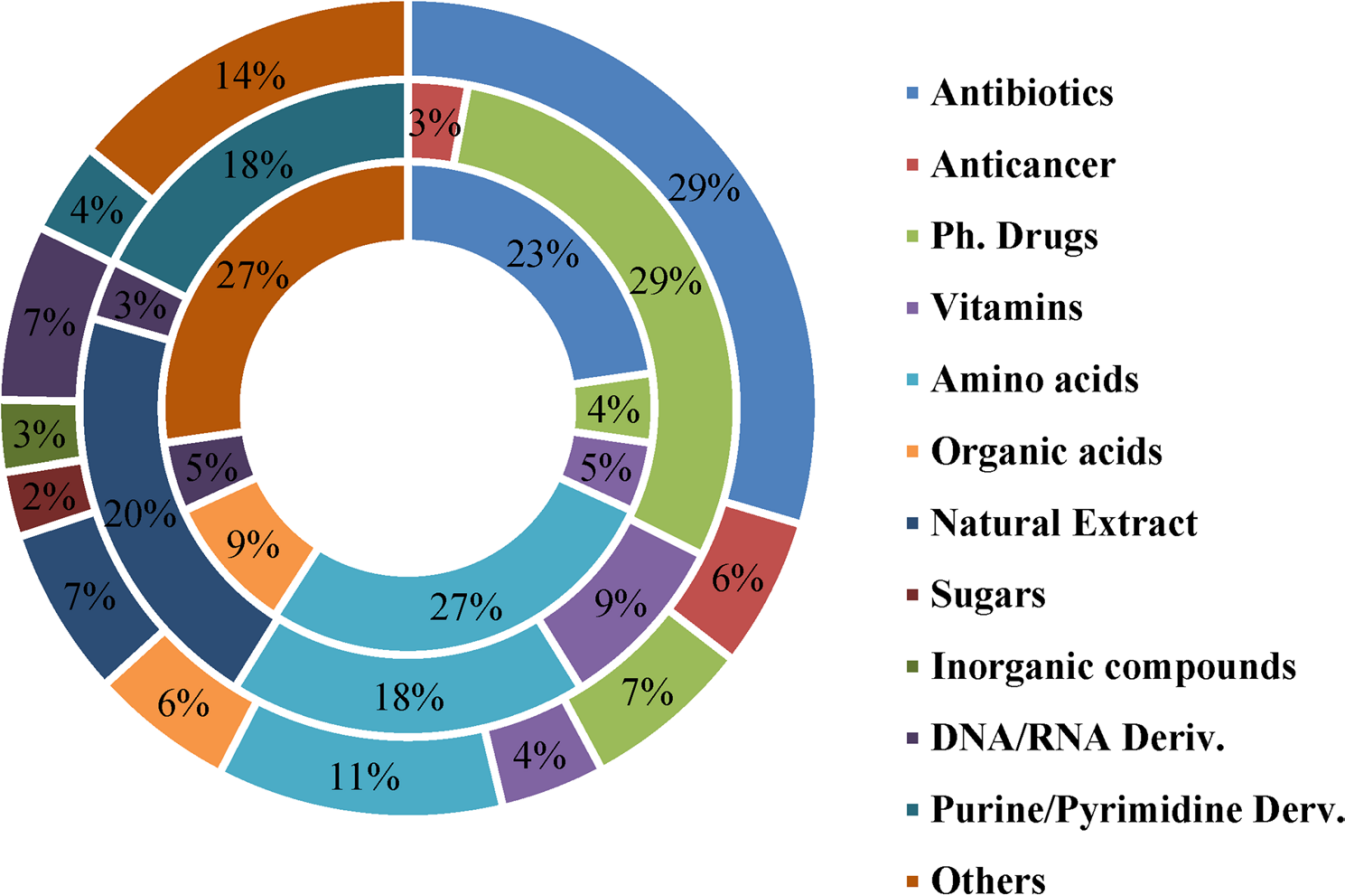
Categorization of obtained drugs with potential anti mycobacterial activities.Obtained drugs were categorized according to their nature/function into different groups. DEG showed the largest number of drugs followed by OGEE then VFDB. The outer doughnut represents DEG, the middle doughnut represents OGEE, and the inner doughnut represents VFDB

A categorization process was implemented on obtained drugs which was based on classifying them according to its nature/function. Drugs were fashioned into a total of 10 categories: antibiotic, anticancer, pharmaceutical drugs, vitamins, amino acids, natural extract, organic acids, sugars, inorganic compounds, DNA/RNA base derivatives, purine/pyrimidine derivatives, and others. This was true for DEG (12 group) however as for OEGG and VFDB, since a relatively fewer drugs were obtained, they were settled into fewer groups (7 groups for each) as shown in Fig. 3.

DEG targets resulted in a total of 389 drugs, meanwhile OEGG & VFDB laid 34 & 22 drugs, respectively. Drugs obtained by DEG were subdivided into 281 for cytoplasmic targets and 108 for remaining targets.

### MIC pinpointing for potential AMA

A meticulous screening of obtained compounds was executed to select readily available nontoxic compounds; both antibiotics and anticancer drugs were omitted on the base of being already used/tested for the former, and the high cost/possibly hazardous side effects for the latter. Different compounds were selected and submitted to testing their possible MICs such as: propanoic acid, citric acid, tartaric acid, maleic acid, malonic acid, benzoic acid, imidazole, 8-amino theophylline, erythritol, 1,4-dithiothreitol, and D-glutamine as shown in Table 2, isoniazid and rifampicin were also tested for their MIC. Compounds with the lowest MICs were tagged as the most effective among the tested agents.

**Table 2:** MICs of multiple tested compounds

Compound	MIC (µg/ml) against M. smegmatis mc(2)155	MIC (µg/ml) against M. tuberculosis H37Rv I	MIC (µg/ml) against MtS1	MIC (µg/ml) against MtS2	MIC (µg/ml) against MtS3	Solubility/Miscibility with H_2_O
Propionic acid	625	312*	312	625	625	+ 1000 mg/ml
Malonic acid	1000	2000	2000	2000	2000	+ 763 mg/ml
Tartaric acid	1250	2500	2500	5000	2500	+ 1400 mg/ml
Citric acid	2500	5000	5000	5000	10,000	+ 383 mg/ml
Benzoic acid	156* X	156* X	312	156	625	- (434 mg/ml in methanol)
Maleic acid	312*	312*	312	156	312	+ 788 mg/ml
8-amino theophylline	312*	312*	625	625	1250	- (12.8 mg/ml in ethanol)
1,4-Dithiothreitol	5000	5000	10,000	10,000	10,000	+ 50 mg/ml
Imidazole	625	312*	312	625	625	+ 663 mg/ml
Erythritol	5000	-	-	-	-	+ 610 mg/ml
D-Glutamine	2500	2500	1250	2500	5000	+ 41.3 mg/ml
Isoniazid	0.125	0.125	0.125	0.125	0.5	+ 140 mg/ml
Rifampicin	0.3125	0.625	0.625	0.3125	0.625	- (25 mg/ml in methanol)

Multiple compounds were tested for their MIC against both*M. tuberculosis*&*M. smegmatis*; compounds were selected from the 3 databases with the abundance of compounds from DEG. *: indicates the most effective compounds. X: compound was previously studied.

### Pinpointing minimum bactericidal concentration (MBC) for tested drugs

An additional filtration was implemented to reach the optimum compound; only water soluble/miscible compounds with the lowest MIC were picked and preceded to the next step. Only maleic acid and imidazole from 11 tested compounds met these criteria and were further tested for their MBC.

Maleic acid illustrated an MBC of 312 µg/ml (similar to its MIC), while imidazole illustrated MBC of 1250 µg/ml (2 fold its MIC), and so maleic acid was selected for further analysis.

### Illustrating maleic acid effect on human lung cells

**Fig. 4:**
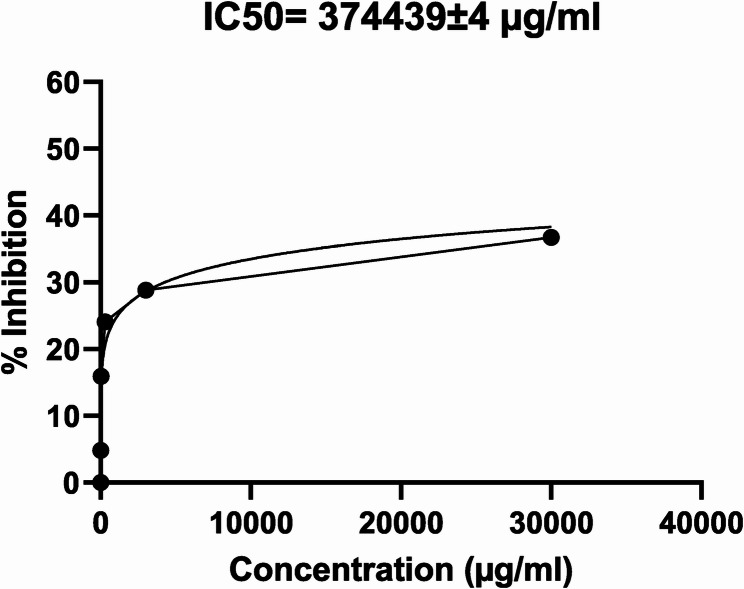
Maleic acid cytotoxicity effect on human fetal lung fibroblast cells (WI-38).Maleic acid effect on lung cells were analyzed using nonlinear regression (curve fit) with variable slope, an IC50 of 374.44 ± 0.004 mg/ml was calculated

Maleic acid illustrated a minimal damage on human fetal lung fibroblast cells (WI-38) with a survivability of ≈ 76% when applying its MIC. The IC50 of maleic acid was identified using GraphPad Prism (Version 9.1.0.221) implementing nonlinear regression (curve fit) with variable slope, IC50 was calculated to be 374.44 ± 0.004 mg/ml (1000X its MIC) as shown in Fig. 4. Selectivity index (SI) was measured for maleic acid using the following equation: SI = IC50/MIC, SI was calculated to be 1200.

### Maleic acid range of activity

Maleic acid was active against both Gram + ve and Gram-ve bacteria but Gram + ve bacteria demonstrated a relatively better sensitivity to maleic acid when compared to Gram-ve bacteria with relatively similar MICs to *M. tuberculosis* as shown in Table 3.

**Table 3:** Maleic acid spectrum of activity

Gram reaction	Organism	MIC (µg/ml)
Mycobacteria	*M. tuberculosis* H37Rv I	312*
Gram positive	*Staphylococcus aureus* ATCC 25,737	625
	*Bacillus cereus* NCTC 2599	312*
Gram Negative	*Escherichia coli* ATCC 25,922	1250
	*Klebsiella pneumoniae* KB700603	625

Maleic acid showed a potential activity against Gram + ve bacteria comparable to its effect on mycobacteria, while also showing to a certain degree probable effect on Gram-ve bacteria

* similar effect

### Induction of potential resistance against maleic acid

**Fig. 5:**
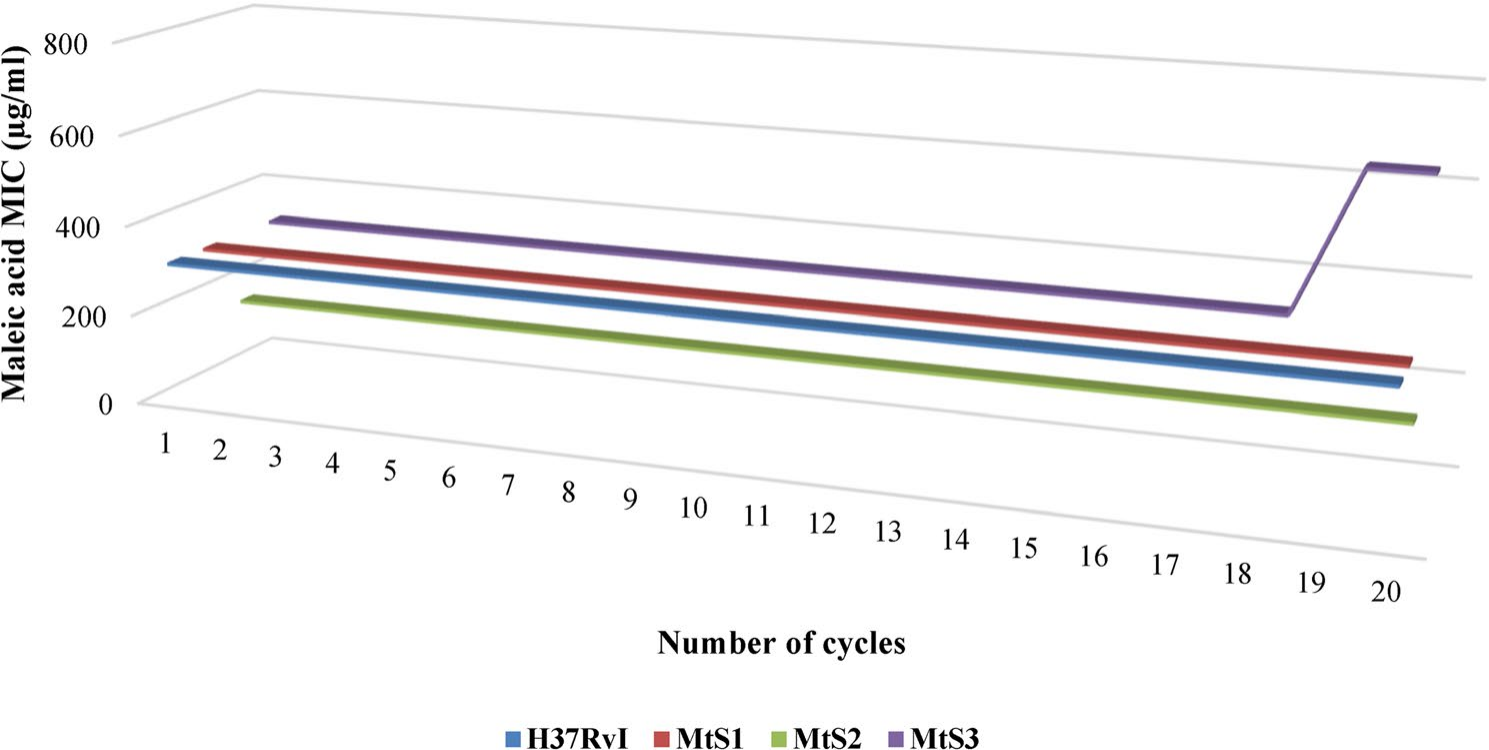
MIC pattern for 20 consecutive cycles.After 20 cycles, both *M. tuberculosis* H37Rv I and clinical isolate MtS1 retained a MIC of 312 µg/ml, clinical isolate MtS2 retained a MIC of 156 µg/ml, while clinical isolate MtS3 illustrated a trifling increase from 312 µg/ml to 625 µg/ml after 18 cycles

Passing *M. tuberculosis* H37Rv I and clinical isolates through 20 consecutive cycles of MIC pinpointing demonstrated a consistent results for H37RvI strain, MtS1, and MtS2, which showed a persistent sensitivity towards maleic acid with a fixed MIC of 312 µg/ml for the first 2 and 156 µg/ml for MtS2, however MtS3 showed a trifling raise in MIC after 18 cycles to be 625 µg/ml (1 fold increase) as shown in Fig. 5.

### Molecular docking of maleic acid against HisC

**Fig. 6:**
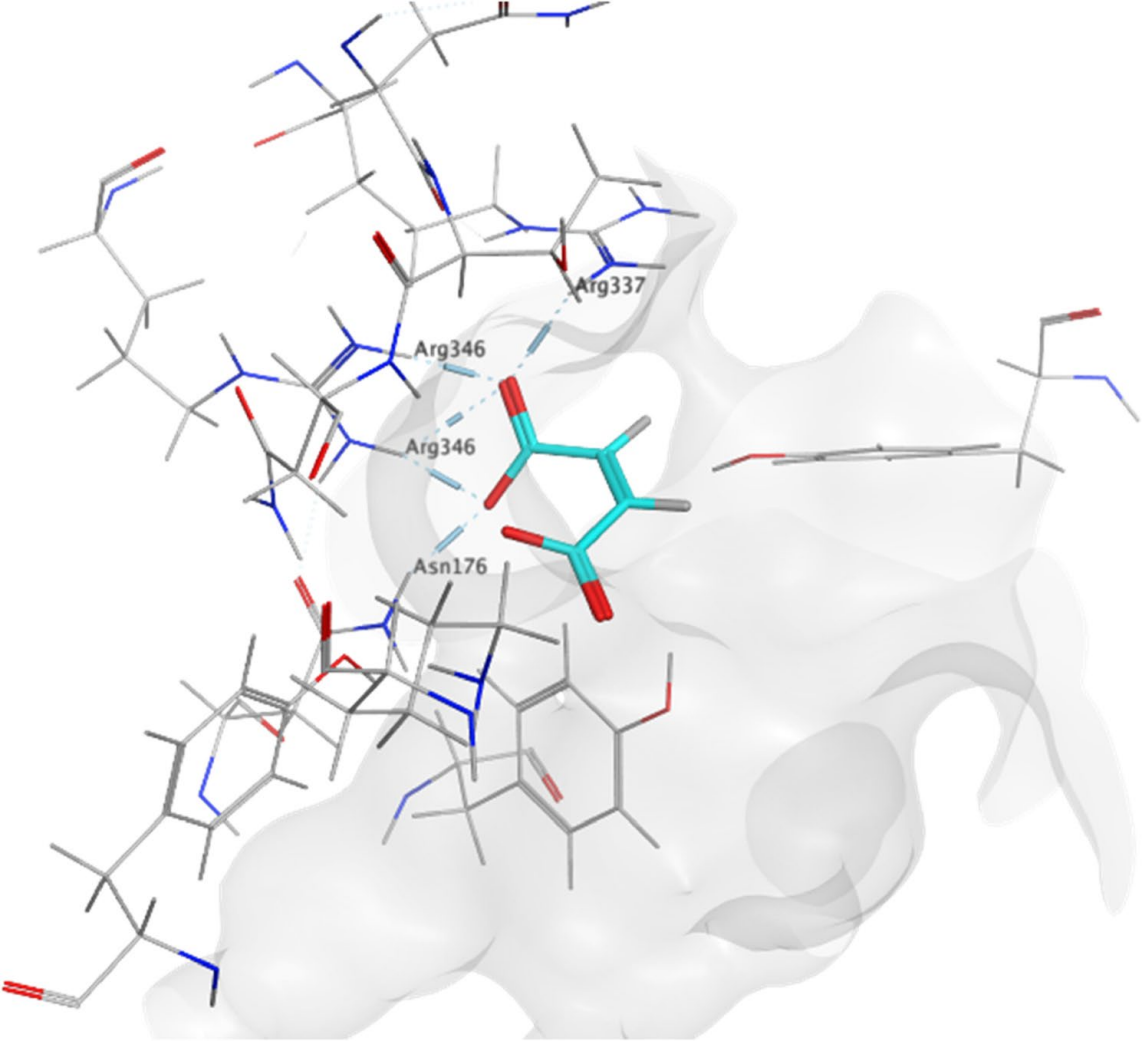
3D-representation of maleic acid against Rv1600 encoded aminotransferase.The results highlight significant conformational flexibility and coordinated atomic motions within the protein-ligand complex, suggesting the presence of functionally important dynamic regions and potential binding sites. (**A**) Variance, (**B**) B-factor values, (**C**) Deformability and (**D**) Covariance matrix

To gain deeper insights into maleic acid and its potential mechanism in antimicrobial therapy, Rv1600 encoded aminotransferase was selected from the literature as a key enzymatic target due to its significant role for the survival and adaptation of *M. tuberculosis* under various stress conditions, including nutrient deprivation and host immune pressure [65]. The docking analysis identified maleic acid as well fitted binder molecule exhibiting a notably high binding affinity (−5.0475 kcal/mol) toward the target enzyme. This compound formed four hydrogen bonds and two electrostatic ionic interactions with critical amino acid residues, including Arg337, Arg346, Asn176 and Lys232 as shown in Table 4; Fig. 6.

**Table 4:**
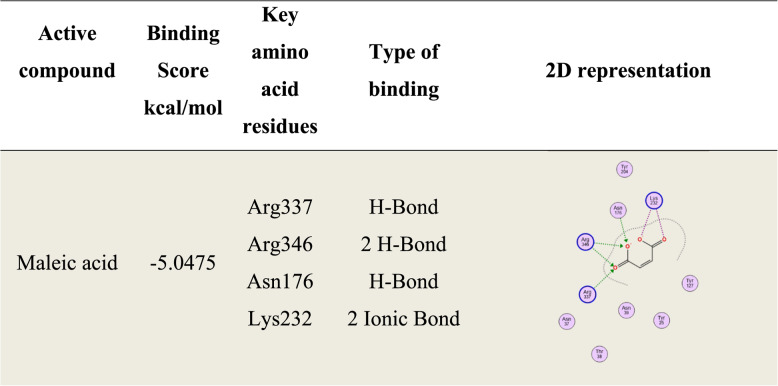
Docking simulation results of maleic acid against Rv1600 encoded aminotransferase (HisC)

### Molecular dynamic simulation

**Fig. 7:**
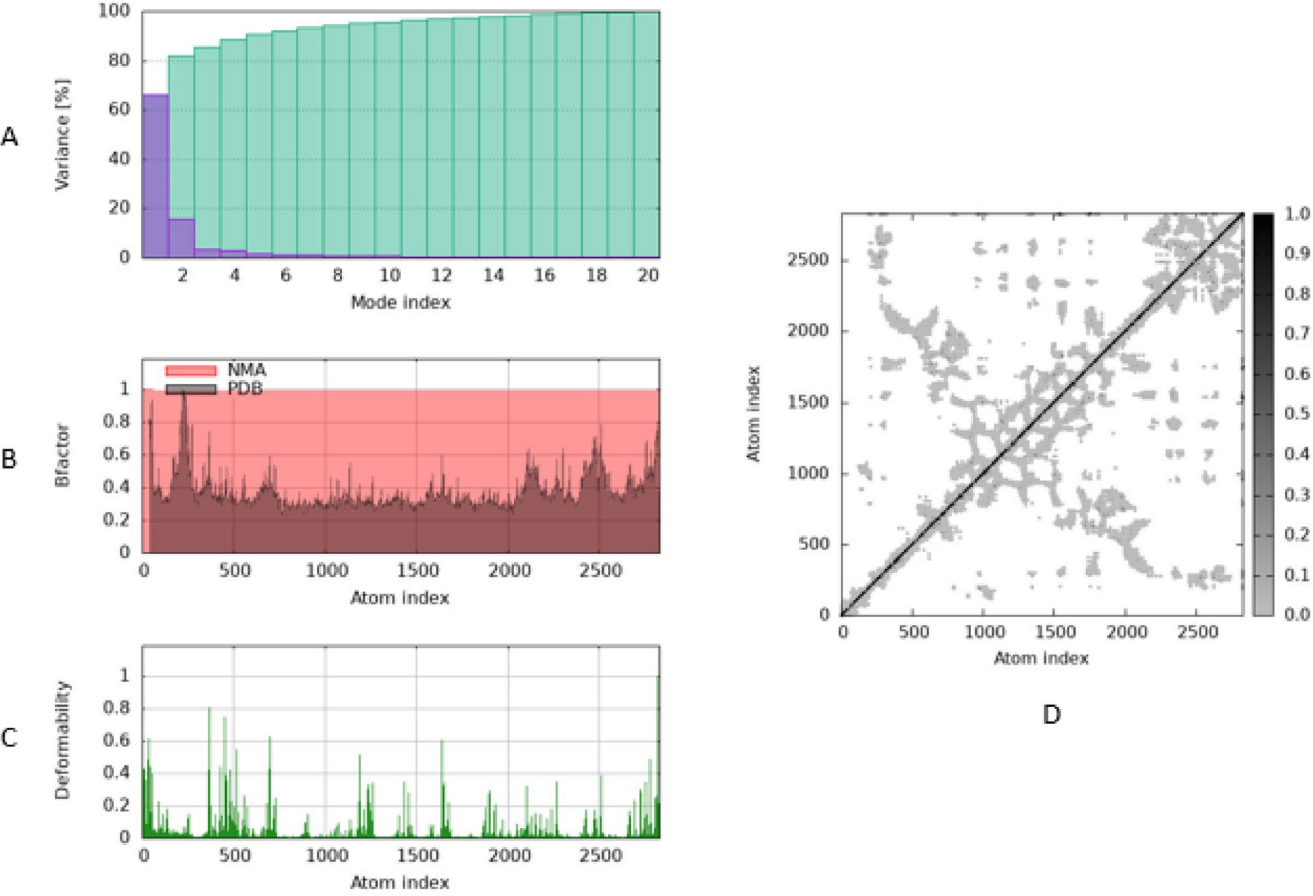
Molecular dynamics simulation of maleic acid/Rv1600 encoded aminotransferase complex by iMODS server.The results highlight significant conformational flexibility and coordinated atomic motions within the protein-ligand complex, suggesting the presence of functionally important dynamic regions and potential binding sites. (**A**) Variance, (**B**) B-factor values, (**C**) Deformability and (**D**) Covariance matrix

The iMODS analysis reveals key insights into the dynamic behavior of the protein-ligand complex. The variance plot shows that the first few normal modes contribute significantly to the overall motion, indicating that the primary conformational changes are captured within these modes. The B-factor graph demonstrates a reasonable correlation between the theoretical (NMA) and experimental (PDB) atomic fluctuations, supporting the validity of the simulation. Peaks in the deformability plot highlight specific regions of the protein with higher flexibility, suggesting potential functional or binding sites. Covariance matrix reveals coordinated atomic motions within the molecule, highlighting flexible regions and dynamic domains. Strong diagonal values indicate self-correlations, while off-diagonal patterns suggest collective movements important for the molecule’s function. Overall, the results highlight significant conformational flexibility and coordinated atomic motions within the protein-ligand complex, suggesting the presence of functionally important dynamic regions and potential binding sites as shown in Fig. 7.

### Preliminary confirmation of selective HisC inhibition

**Fig. 8:**
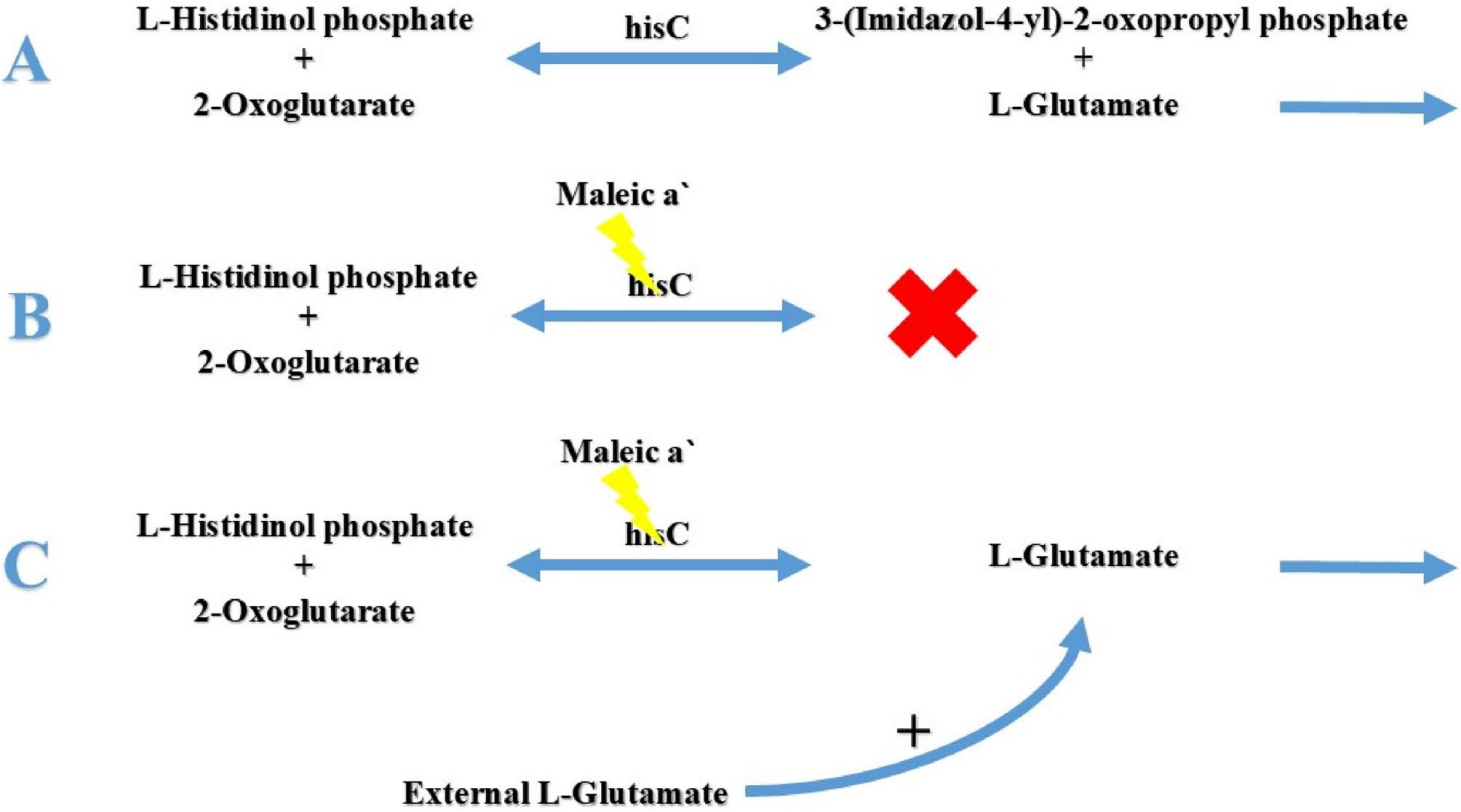
HisC mediated reaction in the absence and presence of maleic acid.This diagram demonstrates the effect of maleic acid on HisC mediated reaction. **A**: Normal enzymatic reaction mediated by HisC, **B**: Illustrates the theorized inhibition of HisC by maleic acid leading to no glutamate formation, hence histidine pathway was terminated, **C**: Upon addition of external Glutamate the pathway was continued even in the presence of maleic acid

**Fig. 9:**
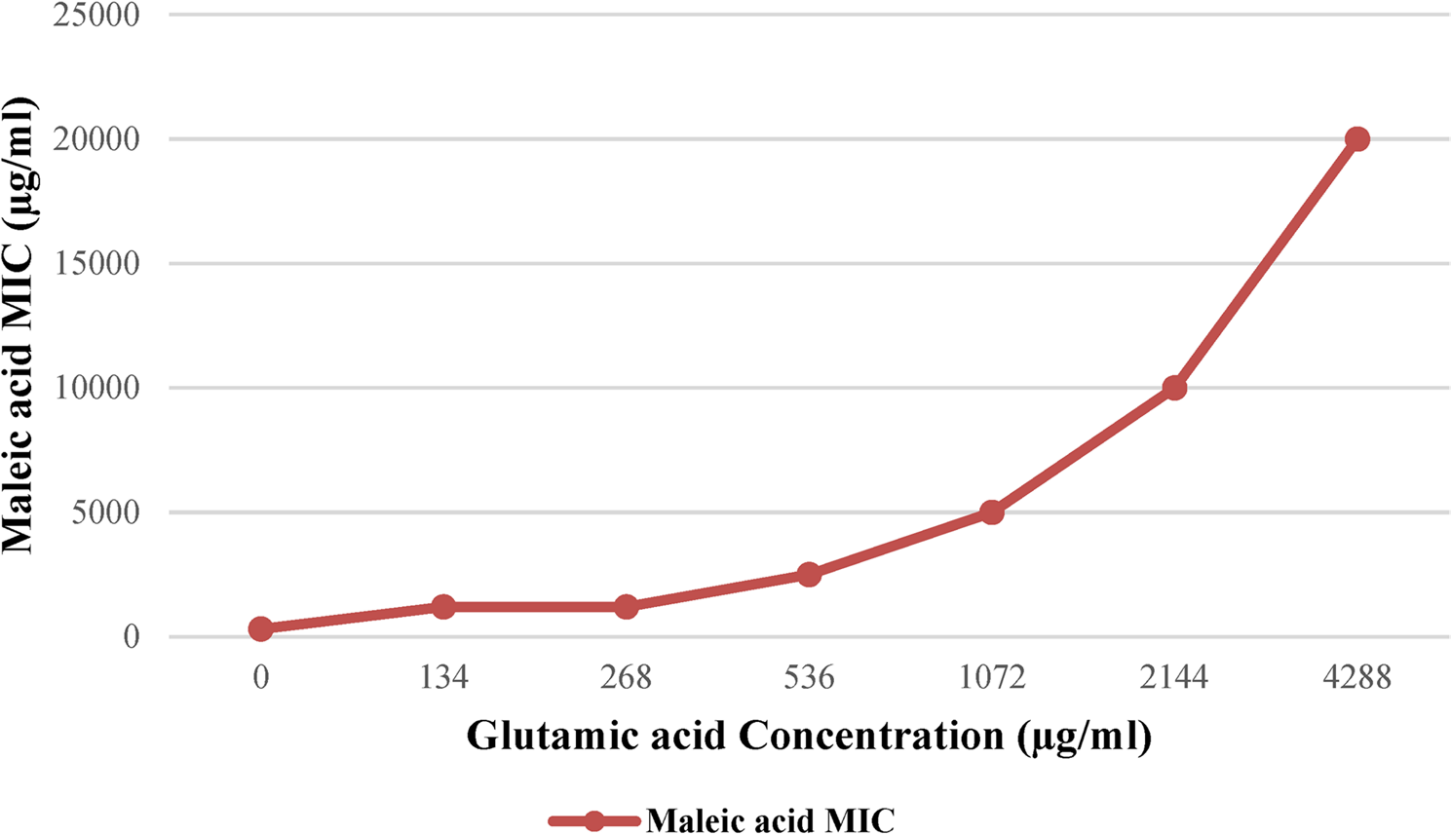
Effect of adding different concentration of glutamic acid on maleic acid MIC against *M. tuberculosis.*Supplementing the reaction media with external glutamic acid resulted in bypassing the theorized effect of inhibiting HisC enzyme by maleic acid, hence retrieving normal histidine cycle and growth of bacteria. Increasing glutamic acid concentration increased MIC steadily till the concentration of 536 µg/ml after which significant increase in the MIC values was observed

The mechanism by which glutamic acid rescues maleic acid inhibition is related to the specific enzymatic reaction targeted by our compound. The HisC enzyme (histidinol-phosphate aminotransferase) which catalyzes the transamination reaction: L-histidinol phosphate + 2-oxoglutarate → 3-(imidazol-4-yl)−2-oxopropyl phosphate + L-glutamate [66]. When maleic acid inhibits HisC, the forward reaction is blocked, reducing glutamate production from this pathway. Glutamate is a critical molecule for *M. tuberculosis*, playing a role in nitrogen metabolism and potentially aiding in acid tolerance [67]. Furthermore, glutamate serves as the primary nitrogen donor for biosynthesis of other amino acids through various aminotransferase reactions [68]. By supplementing exogenous glutamate into the culture media, we bypass the metabolic block caused by HisC inhibition, allowing continued amino acid biosynthesis and bacterial survival as shown in Fig. 8. Supplementing the media with increasing concentrations of glutamic acid successfully led to a corresponding increase in maleic acid MIC as shown in Fig. 9, which indicated a bypass of maleic acid inhibitory activity on *M. tuberculosis* and hence overcoming HisC inhibition, similar approaches were reported in previous literature [51, 52]. The slightest addition of glutamic acid (134 µg/ml) induced a marked 4 fold increase in maleic acid MIC (from 312 µg/ml to 1250 µg/ml) and reduction in its inhibitory activity.

### Potential synergism assessment between maleic acid and standard anti-mycobacterium drugs

**Fig. 10:**
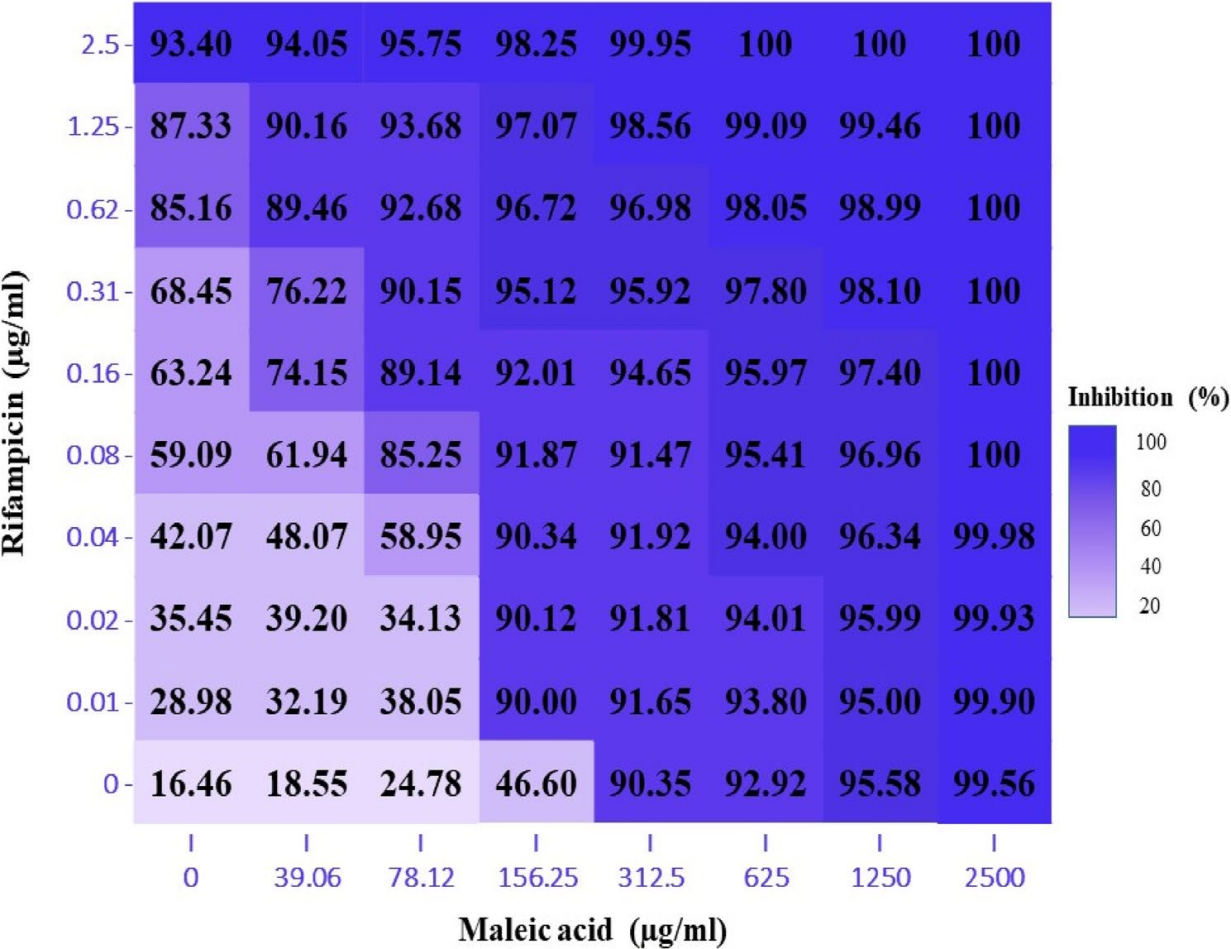
Maleic acid & Rifampicin dose response matrix (inhibition).A graphical illustration of the joint activity of both maleic acid & rifampicin, the Dark blue represents more inhibition while light blue represents less inhibition. Used concentrations were from 39.06 to 2500 µg/ml of maleic acid & from 0.01 to 2.5 µg/ml rifampicin

**Fig. 11:**
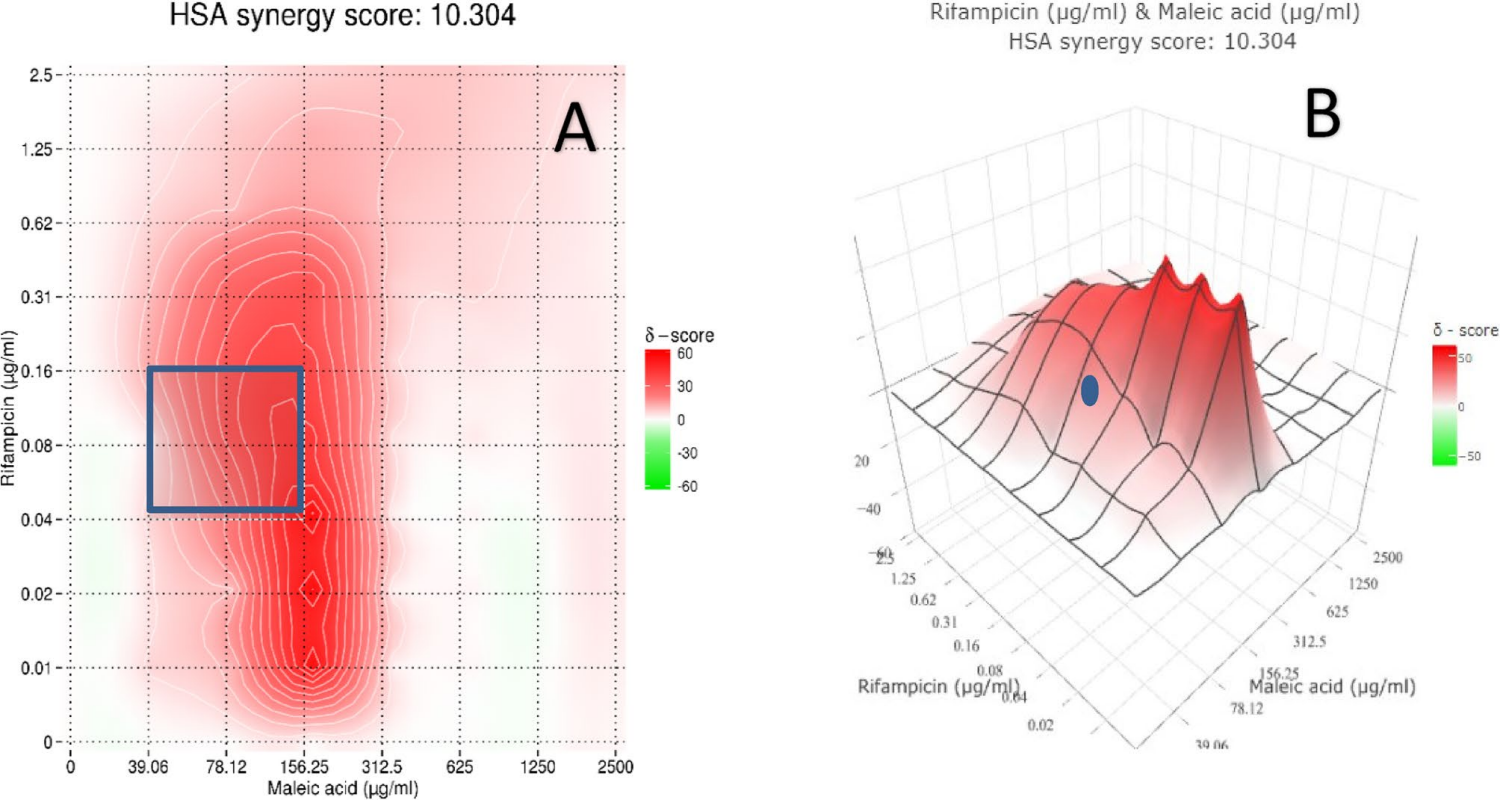
Synergism score between maleic acid and rifampicin.Calculation of synergism score between maleic acid & rifampicin showed a synergy score of 10.303 which is considered synergistic (using HSA method) with maximum synergistic area score was 27.25 as shown in (**A**) lined in blue; the center of such area Align with 78.12 & 0.078 µg/ml for both agents respectively as shown in (**B**) as a blue circle. (**A**): 2D illustration of the 2 agents’ joint activity. (**B**): 3D illustration of the 2 agents’ joint activity

Maleic acid coupled with isoniazid showed an additive reaction with ∑FIC = 1. On the other hand, a better results was observed with rifampicin which illustrated a synergistic activity with maleic acid showing ∑FIC = 0.375 (< 0.5). Synergistic activity between the 2 agents was further confirmed by results obtained from SynergyFinder which constructed a dose response matrix between the 2 agents as shown in Fig. 10, which was used to compute synergism. Analysis demonstrated a synergy score of 10.303 (> 10) and henceforth a synergistic action was confirmed as shown in Fig. 11.

### In-vivo determination of anti-mycobacterial activity of selected agents

**Fig. 12:**
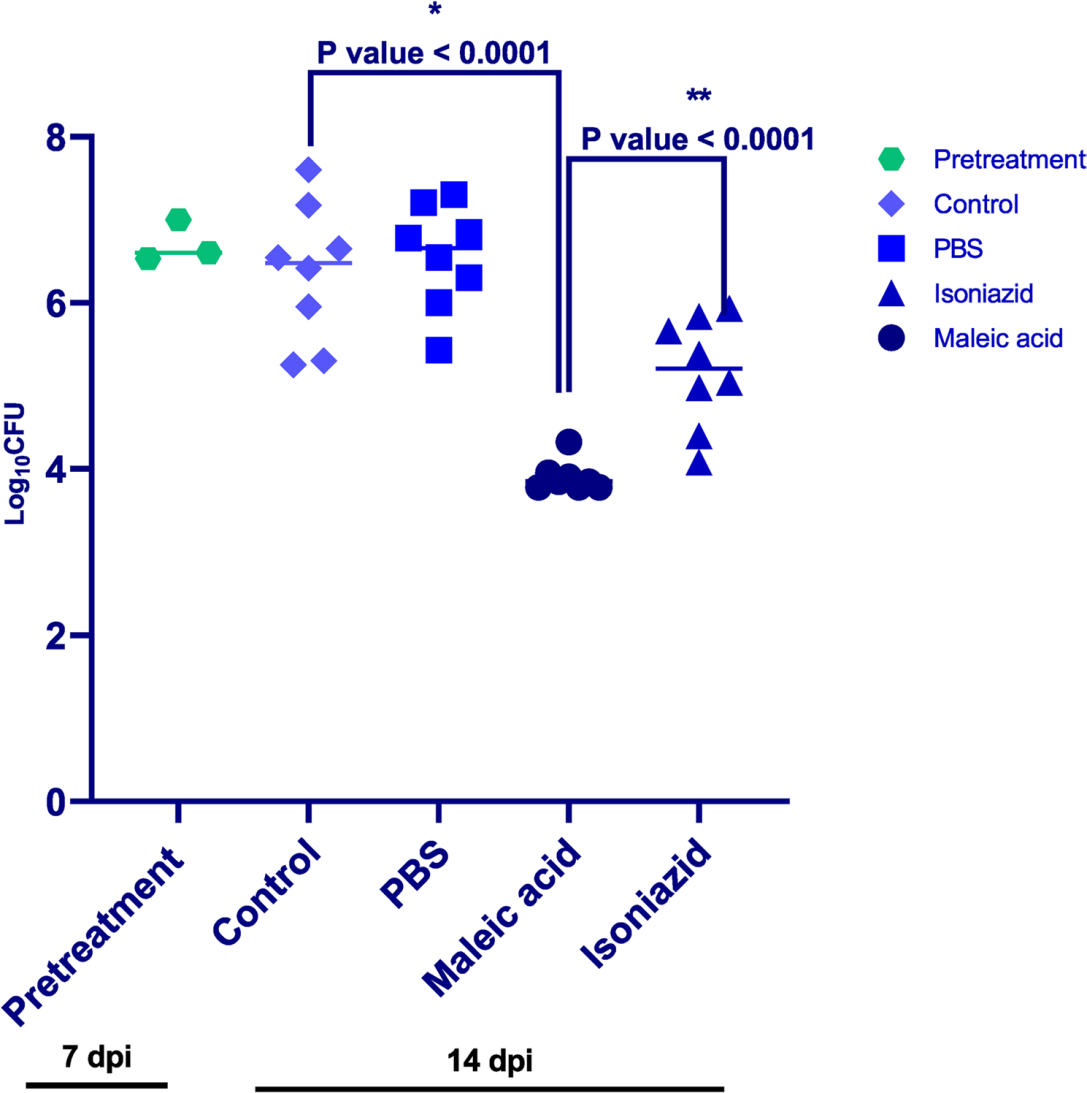
Lung bacterial counts among various animal model groups.The plot of Log_10_ CFU of each group illustrated a significant difference between maleic acid and both control groups and PBS with P value < 0.0001 (*). In the same time comparison of maleic acid efficacy to isoniazid also showed a significant difference with P value < 0.0001 (**). Pretreatment group represents Log_10_ CFU of mice lungs on day 15 before initialization of treatment. dpi: days post infection

After treatment protocol conclusion (14 Days post infection); obtained CFU from various mice groups homogenized lungs were subjected to analysis utilizing GraphPad Prism 9.1.221, unpaired t-test and one-way ANNOVA was utilized to detect significant difference between various groups. Mice that were administered maleic acid showed ≈ 3 log_10_ CFU reduction when compared to both control groups (P value < 0.0001) and ≈ 2 log_10_ CFU reduction against group receiving isoniazid (P value < 0.0001) which were considered significantly different and represented a better in-vivo activity of maleic acid when compared to isoniazid specially after only 1 week of treatment and at a relatively low dose of 6 mg/kg. It’s worth mentioning that isoniazid effect was significantly different from control group (P value < 0.0067) as shown in Fig. 12.

### Histopathological analysis of lung tissue from various treatment groups

Microscopic examination of pulmonary tissue samples revealed:

#### Normal control

Samples showed apparent intact histological features of lung parenchyma with many intact alveolar epithelia with thin inter alveolar septa showing minimal inflammatory cells infiltrates and normal vasculatures. Samples showed intact bronchiolar structures with minimal peribronchiolar inflammatory cells infiltrates.

#### Untreated control

Demonstrated marked diffuse interstitial pneumonia with significant peribronchiolar, inter alveolar wall and perivascular infiltrates with mononuclear inflammatory cells accompanied with bronchiolar epithelium hyperplasia with intraepithelial lymphocytic infiltrates and luminal cellular exudate. Samples showed marked dilatation and hyperemia of pulmonary blood vessels.

#### Maleic acid

**Fig. 13:**
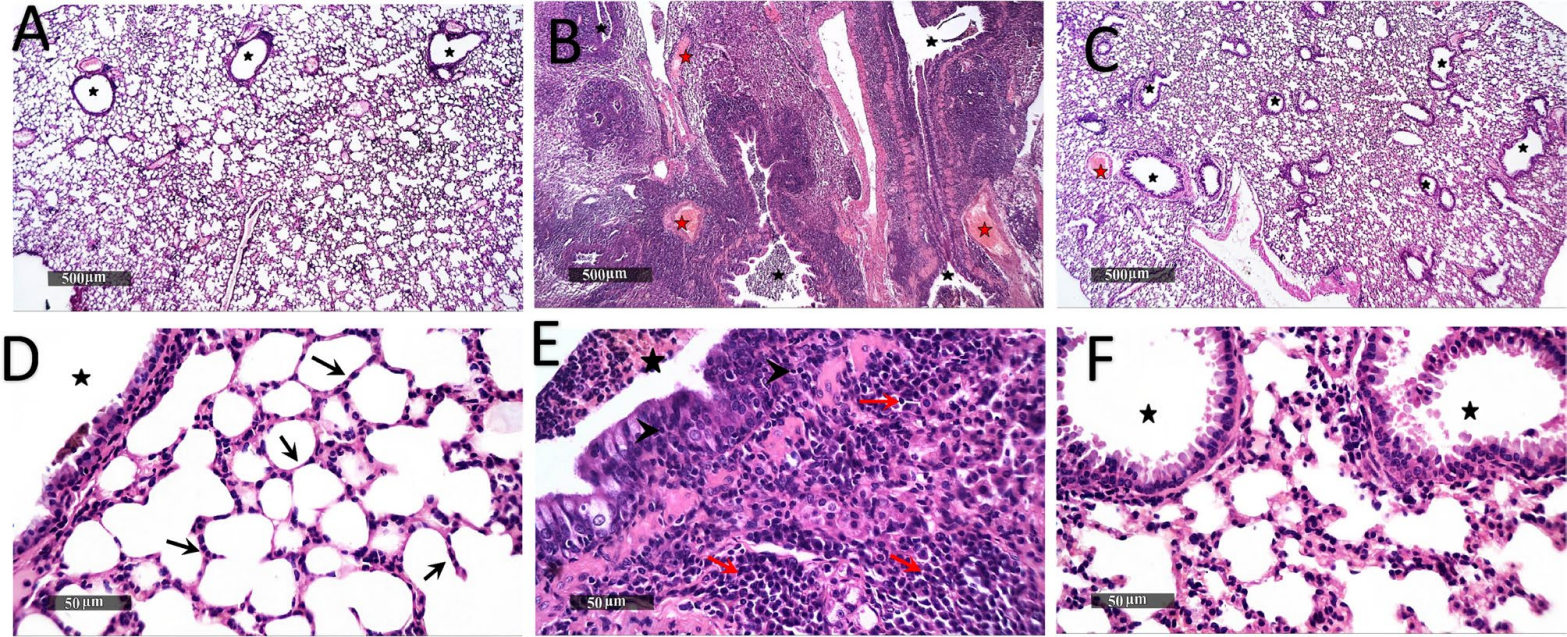
Histopathological analysis of Lung samples.The microscopic examination of lung tissue from normal control group (**A** &** D**) showed intact histological features of lung parenchyma with Intact bronchiolar structures (star) (**A**) with many intact alveolar epithelium with thin inter alveolar septa (arrow) showing minimal inflammatory cells infiltrates and normal vasculatures (**D**). while untreated group samples (**B**, **E**) demonstrated marked diffuse interstitial pneumonia with significant peribronchiolar, inter alveolar wall and perivascular infiltrates with mononuclear inflammatory cells (red arrow)(**E**) accompanied with bronchiolar epithelium hyperplasia with intraepithelial lymphocytic infiltrates (arrow head) (**E**) and luminal cellular exudate (black star)(**B**), and marked dilatation and hyperemia of pulmonary blood vessel (red star)(**B**). Maleic acid group samples (**C**, **F**) illustrated better organized morphological features than untreated group samples with mild records of congested vasculatures (red star)(H) and minimal inflammatory infiltrates records with somewhat intact bronchiolar structures (black star)(**F**)

Samples showed evident protective efficacy and better organized morphological features compared to untreated group with mild records of congested vasculatures and minimal inflammatory infiltrates records. As shown in Figure 13.

## Discussion

In our work, the fruitfulness of utilizing online databases and tools to scavenge for drug targets and potential drugs was evaluated and proved to relatively boost the process of novel AMA discovery in terms of time and money in comparison with traditional methods of either modifying existing agents or synthesizing novel one; all of which possess a high toll in terms of money, time, labor and hazards [69]. Computational guided scavenge for novel drugs and targets not only illustrated numerous databases which can be utilized for uncovering novel drugs, but also offers diverse adjustable different approaches to filter for the optimum targets/drugs (according to individual prospective/target). Among the 3 selected databases DEG showed the highest potential with largest number of targets and subsequently highest number of potential drugs; not only it was more productive but it was also more reliable since each presented protein targets were supplemented with its subcellular localization, nucleotide size & sequence, protein size & sequence, research article proving its essentiality, its coding nucleotide sequence position on leading/lagging strand, KEGG enrichment code, GO enrichment, and EggNOG enrichment (wherever available). While other researchers suggest starting the sequential filtration process with the organism whole genome obtained wither from KEGG [11, 27] database or NCBI [20], we found out that starting with essential targets databases (DEG & OGEE) cut the filtration process short, excludes additional steps, and put the focus on next steps. The filtration steps were selected to reach the most optimum results; exclusion of *homo sapiens* homologous proteins was checked to exclude any unnecessary interaction with human targets or proteins; essentiality testing was to accentuate that inhibiting such targets would kill/stop *M. tuberculosis* proliferation; the selection of cytoplasmic targets since as before mentioned [25] was the main targets for developing/discovering novel antimicrobial (while secreted or external targets were for vaccine development); targets preservation was essential in order to validate targets universality among *Mycobacteria* species.

While the obtained final list of drugs from each database held a big degree of similarity to each other (with DEG maintaining the largest pool of drugs) it was necessary to test at least 1 unique drug from VFDB & OGEE. However after meticulous screening and classification of obtained drugs a further filtration and exclusion process was conducted to reach a more optimum drug lists; hence the following drugs/drug groups were omitted: any drug that was already used/tested against *M. tuberculosis* which included the vast majority of antibiotics, anticancer drugs since they already possess serious side effects and toxicity, any drug that was known to be toxic, and any drug that was of unknown characteristics (Physical or chemical) or that was unavailable.

After MIC pinpointing it was obvious that organic acids held potentials as AMA with maleic acid being the most prominent as it illustrated the lowest MIC (312 µg/ml) against *M. tuberculosis* different strains. Even though this MIC might seem relatively high compared to standard antibiotics (isoniazid, and rifampicin) it was in consistence with previous reports of organic acids MICs which ranged from 125 to 1250 µg/ml [52, 70–73].

MBC: MIC ratio for maleic acid was calculated to be 1 which indicated strong bactericidal activity as similarly reported for fosfamycin, vancomycin, and benzalkonium chloride against multiple standard strains of *Listeria monocytogenes* [74]. Another literature reported similar findings for ciprofloxacin against *Klebsiella pneumoniae* ATCC 700,603 and *S. aureus* ATCC 29,213 [75]. In addition maleic acid is relatively a small molecule which was suggested by literature to boost its traversing across cell membranes and hence enhanced efficacy [76]. Antibacterial activity of maleic acid was reported by other studies as: a biofilm inhibitory agent against *Enterococcus faecalis* [77, 78], antibacterial agent against *S. aureus* and antifungal agent against *Candida albicans* [78]. Another study illustrated that a hydrogel combining maleic acid (5000 µg/ml) with chitosan (has native antibacterial activity) was effective against *E. coli* in ocular infections with sound biocompatibility with ocular tissues [79]. Nonetheless in all of these experiments maleic acid was never tested alone as an AMA against *M. tuberculosis* or any other bacteria up till this moment.

Ocular biocompatibility of maleic acid as shown before augmented the notion of its biosafety which was proven by an IC50 on WI-38 cells of 374.44 mg/ml, this represents a sound safety profile since it’s higher than 1000 times the dose delivered to mice (the dose delivered to mice was 10 times its MIC ≈ 3125 µg/ml) and it had SI = 1200 which indicated higher safety of maleic acid since generally a SI > 10 is considered safe and selective [80, 81]. In pharmaceutical industry Maleate salt of drugs are preferred since it increases drug stability and it’s biocompatible in oral dosage forms such as Enalapril, Methergine, and Bromocriptine tablets [82] or as a salt for bedaquiline (Novel drug for XDR-TB) which showed better stability and less polymorphism when compared to other salts such as tartrate, lactate, fumarate, and malate among others [83, 84]. Another study illustrated that maleate salts had a comparable bioavailability and activity to fumarate salts of bedaquiline (patented form) [85] the similarity in activity could be attributed to fumarate and maleate being geometric isomers with the same molecular formula (C4H2O4).

Challenging maleic acid against different Gram + ve & Gram-ve bacterium was implemented as a mean of evaluating its activity range. In general, it illustrated a relatively higher activity towards Gram + ve bacteria when compared to Gram-ve, this comes in accordance with the fact that *M. tuberculosis* is a Gram + ve bacteria. It was worth noting that *K. pneumoniae* showed a close MIC to that of *M. tuberculosis* which upon further testing can place maleic acid as a potent antimicrobial agent against respiratory infections. Maleic acid can be tagged as a relatively wide spectrum antimicrobial agent for Mycobacteria and other Gram + ve bacteria primarily & Gram-ve bacteria secondly, this is in accordance with previous reports about maleic acid potential antimicrobial activity [77, 78]. Furthermore it was reported that capping silver nanoparticles with maleic acid induced higher antibacterial activity against both Gram + ve (*S. aureus*,* Bacillus subtilis*, and *Micrococcus luteus*) as well as Gram-ve (*Salmonella setubal*,* Enterobacter aerogenes*, and *Agrobacterium tumefaciens*) bacteria with even more bactericidal activity against Gram-ve bacteria [86].

Maleic acid showed a consistent MIC against the *M. tuberculosis* standard strain and 2 out of the 3 clinical isolates after 20 passages; the only deviation came from the 3rd clinical isolate (MtS3) (which already demonstrated relatively higher MIC) with a mild increase of MIC by 1 fold after 18 passages, this deviation is considered relatively trifling when compared to deviation occurring in isoniazid MIC from 0.06 µg/ml to 20 µg/ml (334 fold increase) as mentioned by [87] after 6 passages, or as reported in [88] that after 13 passage isoniazid MIC increased from ≈ 0.05–0.1 µg/ml up to ≈ 256 µg/ml (2560 fold increase).

HisC enzyme was proven to be a critical enzyme as it’s required for not only histidine biosynthesis but also phenylalanine, tyrosine, tryptophan, and novobiocin, it also augments virulence & survival of *M. tuberculosis* inside macrophage cells via interacting with TLR4 receptor and suppressing the secretion IL-12 and IL-6 cytokines (antibacterial pro-inflammatory mediators) among other abilities [89]. Blocking this enzyme represented a crucial anti-mycobacterial approach that can be potentially accomplished by maleic acid. This was initially confirmed by molecular docking of maleic acid against *M. tuberculosis* HisC enzyme which illustrated high binding affinity (−5.0475 kcal/mol) along with significant conformational flexibility and coordinated atomic motions within the protein-ligand complex, suggesting the presence of functionally important dynamic regions and potential binding sites. Additionally, a preliminary confirmation in vitro experiment was conducted by supplementing the media with excess glutamic acid (main HisC enzymatic reaction product) and hence bypassing enzyme blockade, the increased MIC with increasing glutamic acid concentration suggested that lethal activity of maleic acid due to blocking HisC enzyme and subsequently stopping the formation of glutamic acid was overcome, a similar experiment was conducted by [51, 52] which applied the same principle of supplying the MIC experiment with the product of metabolic reaction of the suggested enzyme, and observe any increase in MIC value which probably means bypassing of inhibitory drug effect on the enzyme and hence indirectly provide a preliminary proof of drug binding to the enzyme, however further confirmation by means of enzymatic assay for example could further prove the selective inhibition.

An elevated standard was considered when deciding on drugs interaction type, while some references define a drug combination as synergistic if their ∑FIC ≤ 0.75 [90], we settled on using ∑FIC ≤ 0.5 as the break point as utilized by [91, 92] and further confirmation was carried out using software based tool (SynergyFinder). The additive effect achieved by maleic acid/isoniazid mixture would allow for concomitant prescription without fear of antagonistic activity. Maleic acid/rifampicin mixture demonstrated ∑FIC = 0.375 which falls in synergistic activity domain and showed similarity to the lowest ∑FIC results obtained by [90] for a mixture of: ofloxacin, rifampicin, and ethambutol. Computed ∑FIC was also lower than that computed for rifampicin/cumene hydroperoxide mixture (∑FIC = 0.45) [93], while falling in the average zone when compared to results obtained by [94] for different rifampicin/carbapenems combinations. Synergistic activity of maleic acid and rifampicin would allow to decrease the dose of rifampicin and hence decrease its side effect.

In in-vivo model, dexamethasone treatment was conducted prior to infection so as to lower mice immunity, facilitate the induction and maintenance of infection, and achieve high CFU/lung [57–59]. The use of drugs at a concentrations ≈ 10 times their MIC against *M. tuberculosis* was a conservative and effective starting concentration in order to achieve at least a 1- log10 CFU reduction since intranasal administration achieve higher bioavailability at the lungs and respiratory epithelia when compared to systemic administration as it allows the drugs to reach directly infection site without the need to cross blood - alveolar barrier as suggested by [62, 63]. Improved decline in log10 CFU (≈ 3 log10 CFU reduction) of maleic acid treated group when compared to control group demonstrated a significant in-vivo activity with better preservation of lung tissues and structure as shown in histopathology analysis. When compared to isoniazid treated group maleic acid showed significantly better efficacy with ≈ 2 log_10_ CFU reduction after only 1 week of treatment and at a relatively low dose of 6 mg/kg which indicated an early in-vivo bactericidal activity that was in accordance with our MBC/MIC value of 1 that also indicated bactericidal activity. adding to a high SI and no mice death in maleic acid treated group, all of which conveyed a sound in-vivo safety. In contrast a study evaluating the in-vivo efficacy of Transitmycin (novel *Streptomyces* sp (R2) secondary metabolite) against *M. tuberculosis* showed a 1 log_10_ CFU reduction compared to control group and no significant difference from isoniazid/rifampicin mix was noticed, moreover the drug compound was toxic at high doses [95]. Isoniazid was selected as the drug to compare to while constructing in-vivo study since it is water soluble and has lower side effects (in contrast to rifampicin). Isoniazid in vivo efficacy was in accordance with previous studies [96, 97]. Enhanced in-vivo activity of maleic acid compared to isoniazid can be partially attributed to differences in doses and to different mechanisms of action against *M. tuberculosis*.

## Conclusion

Computational based scavenge for potential AMA holds great potential with possibility of mining more and more AMA by tweaking search parameters and surveying more protein databases. Among different potential AMA, maleic acid had the lowest MIC/MBC value, showed biocompatibility, displayed strong synergistic action with traditional drugs and showed evident based efficacy in-vivo with augmented mice survivability and lung tissue preservation. This can nominate it to be one of the effective antimycobacterial agents used to treat TB infections after passing sub clinical and clinical evaluations.

## Supplementary Information

Supplementary Material 1.

## Data Availability

The datasets used and/or analyzed during the current study are available from the corresponding author on reasonable request.

## Abbreviations

AMA: Antimycobacterial agents
Gram + ve: Gram positive
Gram-ve: Gram negative
HisC: histidinol-phosphate aminotransferase
*M. tuberculosis*: *Mycobacterium tuberculosis*
*M. smegmatis*: *Mycobacterium smegmatis*
TB: Tuberculosis
PBS: Phosphate Buffered Saline
aa: Amino Acid
